# Outcomes Impacting Quality of Life in Advanced Parkinson’s Disease Patients Treated with Levodopa-Carbidopa Intestinal Gel

**DOI:** 10.3233/JPD-212979

**Published:** 2022-04-05

**Authors:** Norbert Kovács, Lars Bergmann, Marieta Anca-Herschkovitsch, Esther Cubo, Thomas L. Davis, Robert Iansek, Mustafa S. Siddiqui, Mihaela Simu, David G. Standaert, K. Ray Chaudhuri, Paul Bourgeois, Tianming Gao, Pavnit Kukreja, Francesco E. Pontieri, Jason Aldred

**Affiliations:** a University of Pécs, Medical School, Pécs, Hungary; b AbbVie Inc., North Chicago, IL, USA; c Edith Wolfson Medical Center, Holon, Israel; d Neurology Department, Hospital Universitario Burgos, Burgos, Spain; e Vanderbilt University Medical Center, Nashville, TN, USA; f Kingston Centre, Monash Health, Melbourne, Australia; g Wake Forest School of Medicine, Winston Salem, NC, USA; h Victor Babes University of Medicine and Pharmacy, Timisoara, Romania; i University of Alabama at Birmingham, Birmingham, AL, USA; j Parkinson’s Foundation International Centre of Excellence, King’s College Hospital, London, UK; k King’s College Institute of Psychiatry, Psychology & Neuroscience, London, UK; l Department of Neurology AZ Groeninge, Kortrijk, Belgium; m Sapienza University of Rome, Rome, Italy; n Fondazione Santa Lucia, IRCSS, Rome, Italy; o Selkirk Neurology, Spokane, Washington, USA

**Keywords:** Dyskinesia, health-related quality of life, levodopa-carbidopa intestinal gel, non-motor symptoms, Parkinson’s disease

## Abstract

**Background:**

It is believed that motor symptoms, including dyskinesia, and non-motor symptoms impact health-related quality of life (HRQoL) in patients with Parkinson’s disease (PD), and that improvements in these metrics are correlated.

**Objective:**

Investigate the relationship between HRQoL and measures of PD severity and treatment efficacy, including motor and non-motor symptoms.

**Methods:**

This was a planned investigation of an international, prospective, single-arm, post-marketing observational study of the long-term effectiveness of levodopa-carbidopa intestinal gel (LCIG) in patients with advanced PD. Pearson correlation coefficients (PCC) were calculated for baseline and change from baseline at 12 months between HRQoL and motor and non-motor symptoms.

**Results:**

A total of 195 patients were included. At baseline, HRQoL was moderately positively correlated with Activities of Daily Living (UPDRS II, PCC = 0.44), non-motor symptoms (0.48), and measures of sleep (0.50 and 0.40); all *p* < 0.001. After 12 months of treatment with LCIG, improvements in HRQoL were moderately positively correlated with improvement from baseline in non-motor symptoms (PCC = 0.42), sleep (0.54), and daytime sleepiness (0.40; all *p* < 0.001), and weakly correlated with improvement in dyskinesia signs and symptoms (PCC = 0.23; *p* = 0.011). Improvement in HRQoL was not correlated with improvements in OFF time or dyskinesia time.

**Conclusion:**

Both at baseline and for change from baseline at 12 months, HRQoL was correlated with baseline and change from baseline in dyskinesia, Activities of Daily Living, and non-motor symptoms, including sleep; but not with baseline or change in OFF time.

## INTRODUCTION

Levodopa is the gold standard for managing Parkinson’s disease (PD), but it has a short plasma half-life that can result in motor fluctuations and dyskinesia [1–3]. One solution to address the short half-life of levodopa for patients with advanced PD has been the development of continuous levodopa infusion with levodopa-carbidopa intestinal gel (LCIG). LCIG has been shown in many studies to improve motor and non-motor fluctuations, dyskinesia, and health-related quality of life (HRQoL) in patients with advanced PD [4–7].

Findings from some studies have shown that motor symptoms and dyskinesia have a significant impact on HRQoL [8–10]. However, results in other studies have shown no correlation or only weak correlations [11, 12], still other studies have been limited by methodological and statistical limitations [13–19]. Non-motor symptoms have also been shown to impact HRQoL, suggesting that the effects of treatment on HRQoL may be more complex than improvements resulting from changes in motor symptoms alone [10, 14–16, 18].

**DUO**dopa/Duopa in Patients with Advanced Par-kinson’s Disease—a **GL**obal **OB**servational Study **E**valuating Long-Term Effectiveness (DUOGLOBE)—is an ongoing long-term effectiveness study of the real-world use of LCIG. While the study is still in progress, results from the second interim analysis of the safety and efficacy of LCIG have been published [20]. These data are the basis of the planned analysis reported here. After 12 months, there was a significant (3.9±3.6 h/day; *p*≤0.001) decrease in OFF time, as well as improvements in the signs and symptoms of dyskinesia (Unified Dyskinesia Rating Scale [UDysRS] total score; *p*≤0.001). Non-motor symptoms (Non-Motor Symptoms Scale [NMSS]), measures of sleep (Parkinson’s Disease Sleep Scale [PDSS-2] and NMSS sleep subdomain; both *p*≤0.001), and daytime somnolence (Epworth Sleepiness Scale [ESS] *p* = 0.042) all showed significant improvements [20]. However, changes in Activities of Daily Living (Unified Parkinson’s Disease Rating Scale [UPDRS II]) and motor symptoms (UPDRS III) were not significant. Importantly, scores on the Parkinson’s Disease Questionnaire-8 (PDQ-8) summary index, which measures HRQoL, were improved by –9.0 points (*p* < 0.001), a substantially greater change than the minimum clinically important difference of –5.94 [21]. This report from the second interim analysis of DUOGLOBE examines the correlation of HRQoL with symptoms of PD at baseline and after 12 months treatment with LCIG.

## METHODS

### Study design and treatment

DUOGLOBE is an ongoing 3-year, international, prospective, single-arm, post-marketing observational study designed to assess the long-term effectiveness of LCIG in patients with advanced PD (https://www.clinicaltrials.gov identifier: NCT02611713). This study is being conducted at 55 movement disorders centers in Australia, Belgium, Hungary, Israel, Italy, Romania, Slovenia, Spain, United Kingdom, and the United States. The study was approved by an independent ethics committee/institutional review board at each site. Here we describe a planned analysis based on data from the second interim analysis. The overall design of DUOGLOBE has been published in detail previously and is described briefly below [20].

### Patients

Patients enrolled in the study must have been eligible for LCIG therapy in accordance with the approved local label and reimbursement criteria in the participating country. Exclusion criteria included previous surgery for PD, including deep brain stimulation (permitted in the United States only after a protocol amendment); current treatment with continuous apomorphine infusion; and/or a Mini-Mental State Examination score < 24.

### Assessments

The primary efficacy measure for DUOGLOBE was the change in the number of hours spent in OFF time as reported by the patient for the day prior to the study visit. Other assessments in this analysis included baseline and change from baseline at 12 months in the duration of dyskinesia as reported by the patient; UPDRS part II and part III scores; UDysRS total score and subdomain scores; NMSS total score and subdomain scores, including the sleep subdomain; PDSS-2 total score that measures sleep quality; ESS total score that measures daytime somnolence; and PDQ-8 summary index score. The purpose of this analysis was to assess the correlations between HRQoL (PDQ-8 summary index), and the measures listed above.

### Statistics

**Fig. 1:**
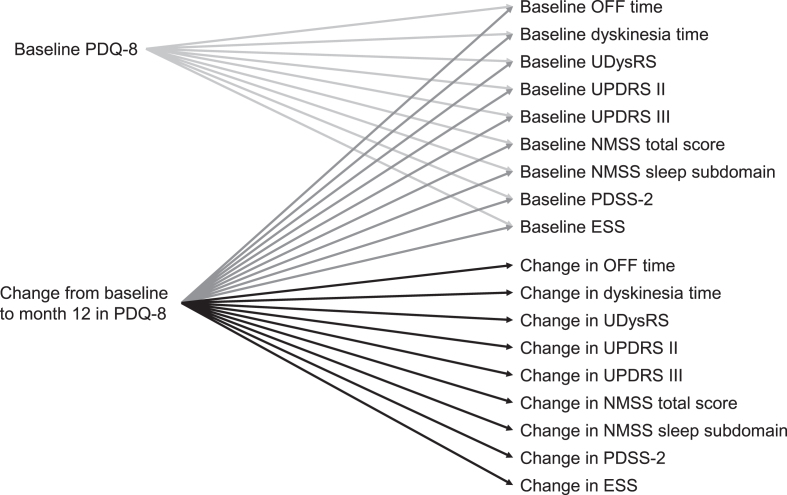
Correlations analyzed. ESS, Epworth Sleepiness Scale; NMSS, Non-Motor Symptom Scale; PDQ-8, 8-item Parkinson’s Disease Questionnaire; PDSS-2, Parkinson’s Disease Sleep Scale-2; UDysRS, Unified Dyskinesia Rating Scale; UPDRS, Unified Parkinson’s Disease Rating Scale.

Demographics and baseline characteristics are presented using descriptive statistics. For this planned analysis, Pearson correlation coefficients were calculated using non-missing data for baseline correlations of HRQoL (PDQ-8) and motor symptoms (OFF time, UDysRS total score, duration of dyskinesia, UPDRS part III), Activities of Daily Living (UPDRS part II), and non-motor symptoms (NMSS total score, NMSS sleep subdomain score, PDSS-2 total score, ESS total score), as well as for change from baseline to month 12 for HRQoL and the above-mentioned outcomes (Fig. 1). Statistical significance was calculated using a two-sided test. All statistical analyses were conducted using SAS version 9.4 (SAS Institute, Cary, NC, USA).

## RESULTS

### Patients

A total of 195 patients, with a mean (standard deviation) age of 70.2 (8.2) years and a mean 11.2 (4.8) years since diagnosis, were enrolled in the study. Baseline characteristics and patient demographics were reported previously [20], and are summarized in Table 1.

**Table 1:** Baseline demographics and characteristics

Characteristic	*n*	LCIG
Age^*^, y	195	70.2±8.2
Male^*^, n (%)	195	120 (61.5)
BMI^*^, kg/m^2^	182	25.9±4.1
PD duration^*^, n (%)
< 10 y	195	94 (48.5)
≥10 y	195	100 (51.5)
Time from diagnosis to LCIG initiation^*^, y	194	11.2±4.8
OFF time^†^, h/day	164	6.0±3.4
Dyskinesia time^†^, h	165	4.1±3.7
PDQ-8 summary index^†^	171	45.1±18.1
UDysRS score^†^	152	33.7±21.1
UPDRS II^†^	173	14.8±7.8
UPDRS III^†^	171	27.6±13.2
NMSS^†^	162	87.9±51.3
NMSS Sleep subdomain^†^	169	16.3±10.5
PDSS-2^†^	171	26.6±11.7
ESS^†^	172	9.8±5.3

All values are mean±standard deviation, unless otherwise specified. ^*^Safety population. ^†^Full analysis set. BMI, body mass index; ESS, Epworth Sleepiness Scale; LCIG, levodopa-carbidopa intestinal gel; NMSS, Non-Motor Symptom Scale; PD, Parkinson’s disease; PDQ-8, 8-item Parkinson’s Disease Questionnaire; PDSS-2, Parkinson’s Disease Sleep Scale-2; UDysRS, Unified Dyskinesia Rating Scale; UPDRS, Unified Parkinson’s Disease Rating Scale.

### Correlation of HRQoL at baseline with baseline measures of PD severity

**Fig. 2:**
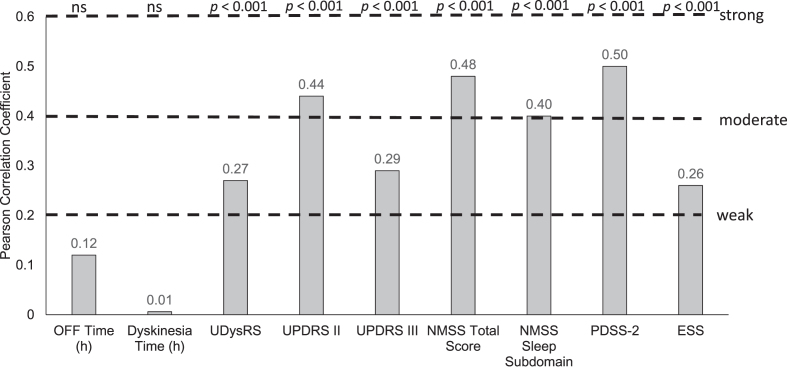
Correlation of baseline parameters with PDQ-8 at baseline. ESS, Epworth Sleepiness Scale; h, hour; NMSS, Non-Motor Symptom Scale; ns, not significant; PDQ-8, 8-item Parkinson’s Disease Questionnaire; PDSS-2, Parkinson’s Disease Sleep Scale-2; UDysRS, Unified Dyskinesia Rating Scale; UPDRS, Unified Parkinson’s Disease Rating Scale.

**Fig. 3:**
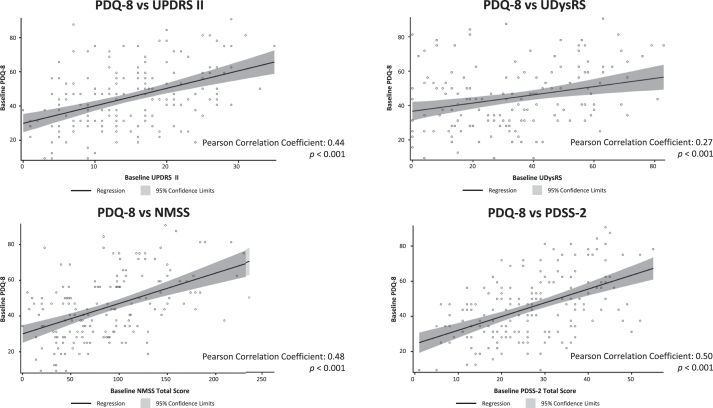
Scatter plots of correlation of PDQ-8 at baseline with baseline UPDRS II, UDysRS, NMSS, and PDSS-2. NMSS, Non-Motor Symptom Scale; ns, not statistically significant; PDQ-8, 8-item Parkinson’s Disease Questionnaire; PDSS-2, Parkinson’s Disease Sleep Scale-2; UDysRS, Unified Dyskinesia Rating Scale; UPDRS, Unified Parkinson’s Disease Rating Scale.

At baseline, there was a moderate positive correlation between HRQoL, as measured by PDQ-8, and baseline Activities of Daily Living (UPDRS II; Pearson coefficient = 0.44), non-motor symptoms (NMSS; Pearson coefficient = 0.48), and sleep (PDSS-2 and NMSS sleep subdomain; Pearson coefficients = 0.50 and 0.40, respectively); all *p* < 0.001 (Fig. 2). PDQ-8 scores were better (lower scores) with better UPDRS II, UDysRS, PDSS-2, and NMSS scores (lower scores; scatter plots shown in Fig. 3). In addition, there was a statistically significant, although weak, positive correlation between baseline UDysRS and baseline PDQ-8 scores (Pearson correlation coefficient = 0.27; *p* < 0.001). Baseline HRQoL was not correlated with baseline OFF time or dyskinesia time.

### Correlation of baseline parameters with improvements in HRQoL

As reported previously [20], mean (standard deviation) scores on the PDQ-8 questionnaire were significantly improved from baseline to month 12 (–9.0±21.6; *p* < 0.001). Correlations between baseline parameters and improvements in PDQ-8 questionnaire scores were statistically significant, though weak. The strongest relationships were negative correlations between absolute changes in PDQ-8 questionnaire scores and baseline PDSS-2 scores (correlation coefficient –0.32; *p* < 0.001), NMSS total score, NMSS sleep subdomain scores (both –0.3; *p* < 0.001), and ESS scores (–0.22; *p* = 0.01), meaning that worse baseline scores correlated with greater improvements in PDQ-8 after initiation of LCIG, making those baseline parameters weak predictors of HRQoL improvement. All other correlations were not statistically significant.

### Correlation of changes in HRQoL with changes in disease parameters after LCIG

**Fig. 4:**
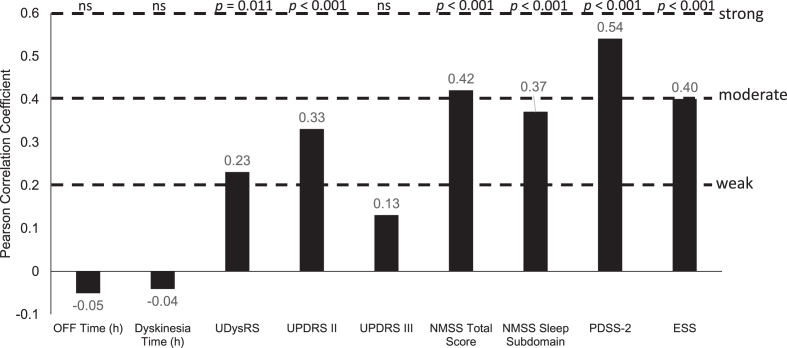
Correlation of change from baseline in efficacy parameters with PDQ-8 change from baseline. ESS, Epworth Sleepiness Scale; h, hour; NMSS, Non-Motor Symptom Scale; ns, not significant; PDQ-8, 8-item Parkinson’s Disease Questionnaire; PDSS-2, Parkinson’s Disease Sleep Scale-2; UDysRS, Unified Dyskinesia Rating Scale; UPDRS, Unified Parkinson’s Disease Rating Scale.

**Fig. 5:**
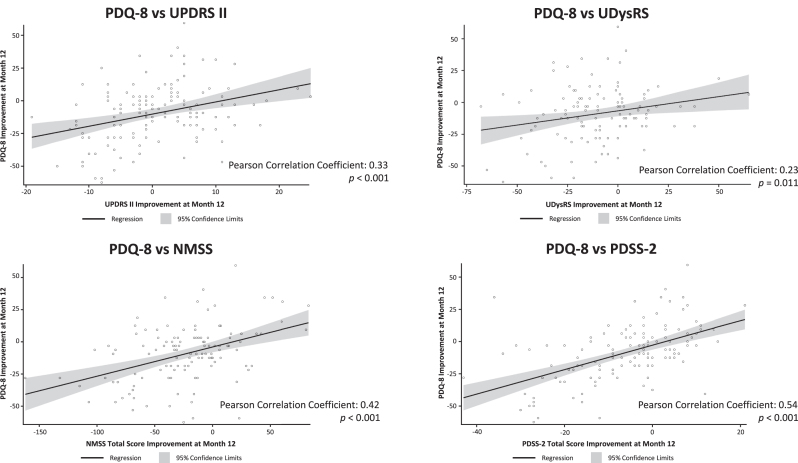
Scatter plots of correlation of change from baseline in PDQ-8 with change from baseline in UPDRS II, UDysRS, NMSS, and PDSS-2. NMSS, Non-Motor Symptom Scale; ns, not statistically significant; PDQ-8, 8-item Parkinson’s Disease Questionnaire; PDSS-2, Parkinson’s Disease Sleep Scale-2; UDysRS, Unified Dyskinesia Rating Scale; UPDRS, Unified Parkinson’s Disease Rating Scale.

After 12-months of treatment with LCIG, absolute changes in PDQ-8 summary index scores were moderately positively correlated with change from baseline in non-motor symptoms (NMSS: Pearson correlation coefficient = 0.42; *p* < 0.001), sleep (PDSS-2, 0.54; *p* < 0.001), and daytime sleepiness scores (ESS, 0.40; *p* < 0.001; Figs. 4 and 5). There were statistically significant weak positive correlations between changes in PDQ-8 summary index scores and changes in UPDRS II scores (0.33; *p* < 0.001), UDysRS scores (0.23; *p* = 0.011), and the NMSS sleep subdomain (0.37; *p* < 0.001; Figs. 4 and 5). Changes in PDQ-8 summary index scores were not correlated with improvements in OFF time or dyskinesia time.

### Safety

The safety results from this study have been previously reported [20]. Serious adverse events are summarized in Table 2. The most frequent serious adverse events were fall (3.1% [*n* = 6]) and urinary tract infection (3.1% [*n* = 6]). There were 13 deaths, of which one death (abdominal obstruction) was believed by the investigator to be related to the study treatment.

**Table 2:** Overview of safety events

Overview	Total (*N* = 195)
SAEs	79 (40.5)
SAEs possibly related to LCIG	23 (11.8)
AEs leading to drug being withdrawn	29 (14.9)
Severe AEs	46 (23.6)
Deaths	13 (6.7)
Deaths possibly related to LCIG treatment^*^	1 (0.5)^†^
MedDRA preferred term	Treatment-emergent SAEs^‡^
Fall	6 (3.1)
Urinary tract infection	6 (3.1)
Hip fracture	5 (2.6)
Pneumonia	5 (2.6)
Abdominal pain	4 (2.1)

^*^As assessed by the study investigator. ^†^Abdominal obstruction. ^‡^Occurring in≥2% of patients by MedDRA preferred term. Data are presented as *n* (%). AE, adverse event; LCIG, levodopa-carbidopa intestinal gel; MedDRA, Medical Dictionary for Regulatory Activities, version 21.1; SAE, serious adverse event.

## DISCUSSION

DUOGLOBE is the first large-scale, prospective, long-term study to simultaneously assess how dyskinesia, motor symptoms, and non-motor symptoms impact HRQoL in patients with advanced PD. Our data show that non-motor symptoms are positively correlated with HRQoL both before treatment and following the start of treatment. There was a moderate positive correlation between measures of non-motor symptoms, including sleep and daytime sleepiness at baseline and PDQ-8 summary index scores at baseline. This is consistent with results from previous studies that have shown that worsening non-motor symptoms, including sleep, significantly worsen HRQoL to a greater extent than do motor symptoms [10, 14–16, 18, 22]. It is evident, therefore, that HRQoL is affected by many factors beyond dyskinesia and motor symptoms alone in patients with advanced PD. Importantly, treatment with LCIG improved both non-motor symptoms and HRQoL [5–7]. In our analysis, improvements in HRQoL in patients treated with LCIG are most strongly correlated with improvements in non-motor symptoms, especially sleep. Further evidence to support this finding comes from the GLORIA registry that indicates that the burden of non-motor symptoms at baseline predicts improvement in HRQoL in patients treated with LCIG [23].

Results from this analysis also demonstrate that there is a moderate positive correlation between Activities of Daily Living (UPDRS II scores) at baseline and HRQoL (PDQ-8 summary index scores) at baseline, which is in line with data presented in previous studies [9–11]. Changes in UPDRS II scores showed a weak positive correlation with changes in HRQoL. Using the short PDQ-8 tool to assess patients with advanced PD may give clinicians a good overview of the effect of PD on HRQoL and thereby indirectly also on Activities of Daily Living. The PDQ-8 questionnaire may also help to identify those patients whose PD is not well controlled with their current medication.

There was a statistically significant, but only weak, positive correlation between the signs and symptoms of dyskinesia (UDysRS total score) at baseline and HRQoL at baseline. Because UDysRS has been introduced more recently and is now increasingly used to assess the anti-dyskinetic efficacy of treatments, this weak correlation was a novel finding [24]. However, although the correlation was weak, treatment with LCIG for 1 year in this interim dataset was associated with significant improvements in the signs and symptoms of dyskinesia (UDysRS total score) [20]; these findings are also consistent with results from an open-label Hungarian registry [25].

In many studies, treatment with LCIG has been shown to improve OFF time in a sustained and highly statistically significant manner, just as it did in this study [4–7]. Surprisingly, there was no correlation between changes in PDQ-8 summary index scores and improvements in OFF time or dyskinesia time while patients were receiving LCIG treatment. Studies of some other treatments for PD have indicated at least weak correlations between dyskinesia, and ON-OFF fluctuations, and HRQoL [8, 11–19]. For example, following deep brain stimulation of the subthalamic nucleus, improvements in HRQoL were influenced by cumulative OFF time, UPDRS III ON/OFF, and UPDRS dyskinesia score in ON time [8]. Our observations with the use of LCIG in this study lead us to conclude that although continuous levodopa treatment usually produces substantial improvements in motor symptoms, the benefits in terms of HRQoL are not derived from these motor effects alone, but rather depend in large part on the effects of LCIG on non-motor symptoms, sleep, and daytime sleepiness.

The present data are insufficient to offer specific recommendations about selecting patients for treatment, but they do suggest that in the population of patients with advanced PD enrolled by the investigators in this study, it is reasonable to expect LCIG to lead to improvements in both motor and non-motor symptoms, and to have an overall beneficial effect on HRQoL.

A strength of this analysis is that data are collected from a large, prospective study in patients with advanced PD who have undergone long-term follow-up and comprehensive assessments of motor complications and non-motor symptoms, although some of these measures could be subject to recall bias. These long-term follow-up data make it possible to evaluate correlations between HRQoL and other motor and non-motor symptoms both at baseline and during treatment with LCIG. However, there are also limitations. Despite the robustness of the DUOGLOBE study, the study is not large enough to conduct meaningful multi-variable analyses to further refine assessments of how these motor and non-motor symptoms might jointly affect HRQoL. Another limitation is that this was an open-label, non-controlled, single-arm study. These results also do not provide insight into the impact that adverse events may have on HRQoL. Given the non-randomized nature of the study and the high probability of potential confounders missing in the data, we abstained from conducting mediation analyses where much stronger assumptions including sequential ignorability or exchangeability, or no unmeasured confounders are required in the analysis but are unlikely to be achieved in this non-controlled, non-randomized observational study. A multi-variable regression analysis was not pursued due to the co-linearity issues from highly correlated motor and non-motor symptoms in the study.

Findings from this analysis have demonstrated that after LCIG treatment in advanced PD, changes in HRQoL are positively correlated with changes in Activities of Daily Living as measured by UPDRS II scores, non-motor symptoms, and sleep. These observations point to the importance of considering effects on non-motor features when evaluating the effectiveness of treatments for PD. The safety findings reported in the 1-year interim analysis of the DUOGLOBE study [20] were consistent with the established safety profile of LCIG.
